# Spatial attention in written word perception

**DOI:** 10.3389/fnhum.2014.00042

**Published:** 2014-02-10

**Authors:** Veronica Montani, Andrea Facoetti, Marco Zorzi

**Affiliations:** ^1^Department of General Psychology, University of PaduaPadua, Italy; ^2^Neuropsychology Unit, “E. Medea” Scientific Institute, Bosisio PariniLC, Italy; ^3^IRCCS San Camillo Neurorehabilitation Hospital, Venice-LidoItaly; ^4^Center for Cognitive Neuroscience, University of PaduaPadua, Italy

**Keywords:** visual word recognition, visuo-spatial attention, spatial cueing, reading aloud, phonological decoding, attentional focusing

## Abstract

The role of attention in visual word recognition and reading aloud is a long debated issue. Studies of both developmental and acquired reading disorders provide growing evidence that spatial attention is critically involved in word reading, in particular for the phonological decoding of unfamiliar letter strings. However, studies on healthy participants have produced contrasting results. The aim of this study was to investigate how the allocation of spatial attention may influence the perception of letter strings in skilled readers. High frequency words (HFWs), low frequency words and pseudowords were briefly and parafoveally presented either in the left or the right visual field. Attentional allocation was modulated by the presentation of a spatial cue before the target string. Accuracy in reporting the target string was modulated by the spatial cue but this effect varied with the type of string. For unfamiliar strings, processing was facilitated when attention was focused on the string location and hindered when it was diverted from the target. This finding is consistent the assumptions of the CDP+ model of reading aloud, as well as with familiarity sensitivity models that argue for a flexible use of attention according with the specific requirements of the string. Moreover, we found that processing of HFWs was facilitated by an extra-large focus of attention. The latter result is consistent with the hypothesis that a broad distribution of attention is the default mode during reading of familiar words because it might optimally engage the broad receptive fields of the highest detectors in the hierarchical system for visual word recognition.

## INTRODUCTION

Visuo-spatial attention is likely to be engaged at many levels of the process of recognizing printed word (McCandliss et al., 2003), but despite many studies investigating this issue the literature does not offer a clear and uncontroversial picture. Several different manipulations of attention have been used to investigate whether word processing is automatic or whether it requires some engagement of attention and, in the latter circumstance, what kind of reading sub-processes consume attention resources. We review below the previous literature and then present a new study examining the involvement of spatial attention in visual word perception, more specifically how the latter is modulated by focusing attention on the target stimulus.

The most cited evidence for the automaticity of word reading is the Stroop effect (for a review see MacLeod, 1991). Longer reaction times (RTs) in naming the ink color of words that convey incongruent color names is usually taken as a demonstration of automatic processing up to the word meaning, thereby suggesting that suppression of word reading is difficult or even impossible (e.g., Neely and Kahan, 2001; Brown et al., 2002). Nevertheless, automatic word processing in Stroop tasks can be moderated by attentional manipulations, as shown by the finding that focusing spatial attention on a single letter of the word can reduce the magnitude of the Stroop effect (e.g., Stolz and Besner, 1999; see also Lachter et al., 2004, 2008).

Another way to investigate the automaticity of word reading is to assess whether it can proceed in parallel with another task. To this aim, some studies have used the psychological refractory period (PRP) paradigm (Pashler, 1994; Johnston et al., 1995), which requires to perform two tasks in rapid succession. When the time interval between the two tasks is long, the two tasks are performed without interference, while RTs for the second task increase sharply when the time interval is short (i.e., PRP effect). McCann et al. (2000) concluded that orthographic-lexical processing needs central attention, whereas Cleland et al. (2006) found exactly the opposite. Other studies, using the locus-of-slack logic, provide evidence that phonological recoding requires central attention while earlier visual-orthographic processing can automatically proceed (Reynolds and Besner, 2006; O’Malley et al., 2008). Lien et al. (2008) used the PRP paradigm in combination with the recording of event-related potentials (ERPs). They assessed the amplitude and latency of the N400 wave elicited by words that were semantically related or unrelated to the context, as well as the amplitude and latency of the P300 wave elicited by high or low frequency words (LFWs). Overall, their conclusion was that neither semantic nor lexical processing can proceed without attention (but see Rabovsky et al., 2008). Converging evidence regarding the role of attention in word reading is also provided by studies on mindless reading (e.g., Reichle et al., 2010; Schad et al., 2012).

Posner’s spatial cuing paradigm (Posner, 1980) allows to direct attention to a particular position in visual space and to assess the consequences of processing a target stimulus at the attended vs. unattended location. In the context of written word perception, orienting spatial attention away from the target should be detrimental if word processing requires attention. However, the studies using variants of this paradigm have produced inconsistent results. Some studies have reported that biasing spatial attention with a cue either at the beginning or at the end of a letter string has a stronger influence on pseudoword (PWs) than on word reading (Sieroff and Posner, 1988; Givon et al., 1990; Auclair and Siéroff, 2002), thereby suggesting that the lexical status of the string can influence the distribution of attention. Other studies, however, reported that the cuing effect was not modulated by the type of string. For example, McCann et al. (1992) found faster lexical decision latencies at the cued position for both words and PWs that were presented above or below the fixation point. Similar results were found using left or right parafoveal presentation (Nicholls and Wood, 1998; Ortells et al., 1998; Lindell and Nicholls, 2003). Finally, a lack of cueing effect was reported by Ducrot and Grainger (2007) using a perceptual identification paradigm with target words appearing left or right of a central fixation point and using a string of hash marks as spatial cue. In valid trials the cue matched the target both in location and spatial extent, while in the neutral condition the hash marks covered both possible locations of the target. When the target was presented in central vision, with fixation either on the first or on the last letter, little or no effects of spatial cueing were found. However, it is important to note that the absence of invalid trials might have influenced the latter results.

Familiarity of the stimulus is typically manipulated through the frequency or the lexicality of the string (e.g., Monsell et al., 1989; Allen et al., 2005). A different approach was adopted by Risko et al. (2011), who used repetition to manipulate familiarity and combined it with spatial cueing in the context of a word naming task. They found that in the repetition condition (i.e., when the word was repeated numerous times throughout the experiment) the cueing effect was smaller than in the no repetition condition (i.e., when the word was presented a single time). This finding is in line with the idea that familiar items place less demands on spatial attention. Moreover, the study of Risko et al. (2011) offers an explanation of the inconsistent findings on the automaticity of reading, because the findings using the Stroop task may reflect the fact that stimulus repetition reduces spatial attentional requirements.

In summary, the studies reviewed above suggest that attention is flexibly used in visual word processing. This is also consistent with the finding of individual differences in the automaticity of visual word recognition that largely depend on reading skills (Ruthruff et al., 2008) and presumably on reading experience (Siéroff and Riva, 2011). In contrast to the idea of fully automatic processing that is highlighted by the Stroop task, the engagement of attention seems a necessary requirement in order to process visually presented words.

### SPATIAL ATTENTION IN MODELS OF READING ALOUD

Beginning readers need to learn a system for mapping between visual symbols and sounds (Ziegler and Goswami, 2005). Simple visual features are combined to form detectors of letter shapes (Dehaene et al., 2005; Zorzi et al., 2013) and letters are then organized into higher-order units that map onto sounds (Perry et al., 2007, 2013). Indeed, phonological decoding is thought of as *sine qua non* for reading acquisition (Share, 1995). Repeated exposure to the printed material and the ability to recognize words through phonological decoding progressively leads to the development of orthographic representations of whole words (Ziegler et al., 2014, and Di Bono and Zorzi, 2013, for computational models of orthographic learning), with a neural substrate in the occipito-temporal area (i.e., the visual word form area, McCandliss et al., 2003; Glezer et al., 2009; Dehaene and Cohen, 2011). The distinction between phonological decoding (which involves small grain-size units) and recognition of whole words is a prominent feature of dual-route models of reading aloud (e.g., Coltheart et al., 2001; Perry et al., 2007, 2010). Nevertheless, the assumption that reading involves the interaction between two different pathways, one phonological and the other lexical-semantic, is shared by virtually all computational models (e.g., Plaut et al., 1996; Harm and Seidenberg, 2004; for a review see Zorzi, 2005).

In line with the seminal proposal of LaBerge and Samuels (1974), some of these models make specific assumptions on how attention is engaged in the two different pathways. In the CDP+ model (Perry et al., 2007), spatial attention is assumed to be engaged by the phonological pathway during the parsing of letter strings into the constituent graphemes that provide the input to the phonological decoding network (see also Perry et al., 2013). Other models assume a parsing mechanism that can operate on units of different sizes (e.g., letters vs. syllables; Ans et al., 1998) depending on the context. Regardless of the specific details, parsing in all models is thought to rely on focused spatial attention that moves from left to right across the letter string. That is, a top-down search mechanism is used to sweep the spotlight of attention serially over the sub-word units (Vidyasagar, 1999; Vidyasagar and Pammer, 2010).

Several lines of evidence support the hypothesis that the phonological route, rather than the lexical route, requires efficient focusing of visual-spatial attention. Patients with severe neglect dyslexia show preserved lexical-semantic access in reading (Ladavas et al., 1997a,b), suggesting an interaction between the attentional system and the different reading routes. Moreover, several studies have linked developmental reading difficulties to impaired visual-attentional processing mechanisms. Impaired visual-spatial attention has been repeatedly described in dyslexic children (e.g., Facoetti et al., 2005) and adults (Laasonen et al., 2012), in particular for those showing poor non-word reading ability (Cestnick and Coltheart, 1999; Buchholz and McKone, 2004; Facoetti et al., 2006, 2010; Roach and Hogben, 2007; Jones et al., 2008). Non-word reading performance taps the functioning of the phonological route and its impairment is a hallmark of dyslexia across different languages (Ziegler et al., 2003). Dyslexic children perform worse on visual-attention span tasks (i.e., tasks measuring the number of distinct visual elements that can be simultaneously processed at a glance) than normally reading children (Bosse et al., 2007). Moreover, the reading performance of dyslexic children can substantially improve after training visuo-spatial attention (Geiger and Lettvin, 1999; Facoetti et al., 2003; Franceschini et al., 2013) or through a simple manipulation of the physical appearance of the text (i.e., extra-large spacing of the letters) that reduces the demands on focused spatial attention (Zorzi et al., 2012; Schneps et al., 2013). Finally, visual-spatial attention skills in pre-schoolers is predictive of future reading performance (Franceschini et al., 2012).

The aim of this study was to further investigate how visual word processing is modulated by the allocation of spatial attention. Following Ducrot and Grainger (2007), we assessed the effect of a spatial cuing manipulation within a perceptual identification task. Importantly, and in contrast to the study of Ducrot and Grainger (2007), we included an invalid spatial cue condition and we manipulated the lexicality of the stimuli (by including PWs) in addition to familiarity (i.e., word frequency). We predicted that high frequency words (HFWs) should be less influenced by the distribution of attention than LFW, whereas PW should be the most influenced by the attention modulation because phonological decoding places particular demands on the orienting of focused visuo-spatial attention (Perry et al., 2007).

## MATERIALS AND METHODS

### PARTICIPANTS

Twenty undergraduate students from University of Padua participated in the study. Their mean age was 22.85, with range of 18 to 28 years. They were all native Italian speakers and had normal or correct-to-normal vision.

### APPARATUS AND STIMULI

Stimulus presentation was on a 17” CRT monitor connected to a Pentium IV computer running E-Prime 1.1 software (Schneider et al., 2002). Strings were presented in uppercase white letters against a black background in 12-point Courier New font. Participants were seated at a distance of 60 cm from the screen. Each string subtended a visual angle of 4.25°. Two hundred and sixteen eight-letter strings were used as target. Seventy two strings were HFWs (mean printed frequency greater than 33 occurrences per million; Bertinetto et al., 2005), whereas seventy two strings were LFWs (mean printed frequency less than 3 occurrences per million). Finally, seventy two strings were PW obtained by replacing two letters in a set of HFWs (different from those used as targets). In each frequency set, words were 88% nouns, 8% verbs, and 4% adjectives. The target strings were presented in the left or right visual field such that either the last letter or the first letter were aligned with the central fixation point.

In the valid condition, the spatial cue consisted of a string of eight hash marks (########) presented either in the right or left visual field accordingly with the location of the target string. In the invalid condition, the same spatial cue was presented either in the right or left visual field, opposite to the target string. In the neutral condition, the spatial cue consisted of a string of fifteen hash marks, presented centrally and covering both the right and left positions. The central fixation consisted by two vertically aligned central lines with a gap between them (as in Experiment 3 of Ducrot and Grainger, 2007) in order to avoid masking effects.

### DESIGN AND PROCEDURE

**FIGURE 1:**
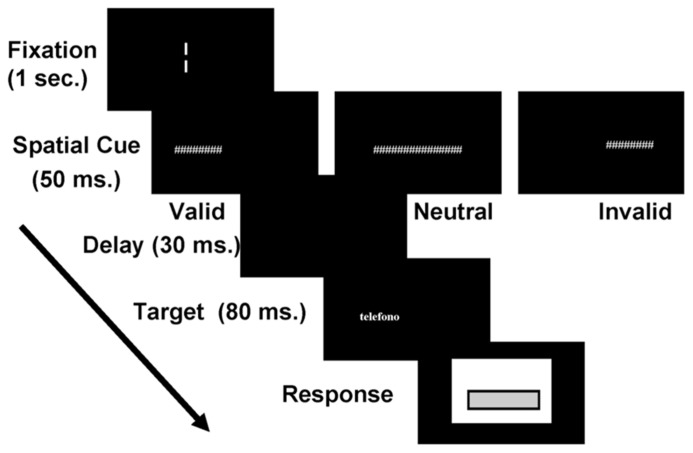
**Experimental paradigm.** Each trial started with the “fixation gap.” After a delay of 1000 ms, the spatial cue appeared for 50 ms. After 30 ms of delay the target string was presented for 80 ms. The response window remained on the screen until participants typed in the perceived string.

Participants had their head positioned on a headrest and they were instructed to avoid eye movements. At the beginning of each trial, the fixation was displayed in the middle of the screen and participants were instructed to fixate the gap. After a delay of 1000 ms, the spatial cue appeared for 50 ms. After 30 ms of delay (i.e., cue-target interval was 80 ms), the target string was presented for 80 ms (**Figure 1**). Then, a window appeared on the screen inviting the participant to type the corresponding string using the computer keyboard.

Every experimental session was divided in two block with a short break between them. During the experiment, target strings were randomly presented such that every string was presented once and their position in the visual field, left vs. right, was randomly chosen such that half of the stimuli were assigned to the left presentation and the other half to the right presentation. The spatial cue condition (valid, invalid, and neutral) was randomly chosen such that each condition had a probability of one third. Therefore, the experiment consisted of three within subjects manipulations: type of string (HFW, LFW, and PW), spatial cue (valid, invalid, and neutral) and visual field (left and right).

## RESULTS

Data were analyzed employing mixed-effect multiple regression models (Baayen et al., 2008) using lme4 package (Bates et al., 2013) and afex package (Singmann, 2013), in the R environment (R Core Team, 2013). Mixed-effects models offer a flexible framework for modeling the sources of variation and correlation that arise from grouped data. In particular, the model fitting procedure takes into account the covariance structure of the data including random effects (for an exhaustive discussion about fixed and random effects, see Gelman, 2005). A great advantage of mixed models, as compared to more conventional methods, is that they do not assume independence amongst observations allowing a wide variety of correlation patterns to be explicitly modeled (Pinheiro and Bates, 2000). Another advantage is that mixed models can deal with the problem of the language-as-fixed-effect fallacy (Clark, 1973). Since it is not possible to make use of systematic sampling procedures both with subjects and items, bringing them as random effects into the model allows controlling better the unexplained by-subject and by-item variances. Overall, mixed models provide insight into the full structure of the data, they have slightly superior power (Baayen, 2008) and finally, they can also be extended to non-normal outcomes.

Response accuracy was computed by counting, for each item, the number of letters correctly reported by the participant. Each letter had to be reported in the correct position in the string to be counted as correct. Nevertheless, the results were virtually identical using a more lenient criterion that did not considered letter position. Note that string-level accuracy was too low for PWs to allow for meaningful analyses. We applied a multiple regression model with a logarithmic link function (Jaeger, 2008) and poisson variance distribution that is appropriate for counts of events in a fixed time window (e.g., Agresti, 2007; Baayen, 2008). Mean accuracies in the different conditions are reported in **Table 1**.

**Table 1:** Mean accuracy (in percentage of correctly reported letters) and standard deviation (in parenthesis) for all conditions in the experiment.

	Left visual field			Right visual field		
String type	Valid	Neutral	Invalid	Valid	Neutral	Invalid
High frequency words	32.81 (17.36)	38.44 (17.37)	29.84 (14.94)	79.01 (12.71)	83.70 (11.53)	76.98 (12.12)
Low frequency words	26.61 (11.91)	24.22 (14.61)	21.46 (12.83)	65.62 (16.00)	66.82 (12.85)	63.28 (9.21)
Pseudowords	23.02 (12.42)	18.07 (7.90)	19.53 (10.25)	49.11 (9.10)	48.80 (8.43)	46.51 (11.14)

Barr et al. (2013) suggested that linear mixed-effects models generalize best when they include maximal random effects structure justified by the design. In our study, this implies the exclusion of the by-item random slopes for type factor because our manipulation of string type implies different items for each level of the type factor. Subsequently, overfitted models (i.e., models with a random structure that caused the model to break) or random effects with no explanatory power (with variance parameters driven to zero or the correlations to +1 or -1) were excluded. Therefore, the final random structure included both by-subject and by-item random intercepts and random variation (random slopes) for the cue factor at the subject level and random variation (random slopes) for the visual field factor at the item level.

The model included three fixed effect and their interactions: *type of string*, *spatial cue*, *visual field*, two way-interactions *type of string* by *spatial cue*, *type of string* by *visual field*, *spatial cue* by *visual field*, and the three-way interaction *type of string* by *visual field* by* cue.* **Table 2** reports random effects of the final model. There was inter-subject variability and it was moderately modulated by the spatial cue effect. Furthermore, the variability in the neutral condition was correlated with the variability in the valid condition (0.79) and it was negatively correlated with the variability in the invalid condition (-0.66). There was inter-stimulus variability modulated by the visual field effect. Importantly, taking into account both these sources of variability, all predictors (fixed effects) considered were significant. **Table 3** reports fixed effect coefficients of the final model (factors were dummy coded with HFW, neutral cue and right visual field as reference levels). Note that the *b* coefficient represents the adjustment with respect to the reference level.

**Table 2:** Random effects of the final model.

Groups	Name	Variance	SD	Corr	
Item	(Intercept)	0.0157	0.1253	–	–
	VF: L	0.1629	0.4037	0.09	–
Sub	(Intercept)	0.0265	0.1629	–	–
	Cue: I	0.0011	0.0325	-0.06	–
	Cue: V	0.0034	0.0583	0.79	-0.66

*Visual field (VF): L = left. Sub: subjects. Cue: V = valid, I = invalid.*

**Table 3:** Fixed effects of the final model.

	Estimate	SE	*z* value	Pr(>|z|)
(Intercept)	1.88	0.05	40.05	0.00
Type: LFW	-0.22	0.04	-5.11	0.00
Type: PW	-0.54	0.05	-11.47	0.00
Cue: I	-0.08	0.04	-2.06	0.03
Cue: V	-0.06	0.04	-1.54	0.12
VF: L	-0.93	0.07	-13.74	0.00
TypeLFW × CueI	0.02	0.06	0.37	0.71
TypePW × CueI	0.03	0.06	0.53	0.59
TypeLFW × CueV	0.04	0.05	0.72	0.47
TypePW × CueV	0.06	0.06	0.98	0.33
TypeLFW × VFL	-0.17	0.06	-1.73	0.08
TypePW × VFL	0.12	0.10	-1.13	0.26
CueI × VFL	-012	0.07	-1.69	0.09
CueV × VFL	-0.07	0.07	-1.03	0.30
TypeLFW × CueI × VFL	0.10	0.16	0.91	0.36
TypePW × CueI × VFL	0.25	0.11	2.23	0.03
TypeLFW × CueV × VFL	0.10	0.10	0.99	0.32
TypePW × CueV × VFL	0.32	0.11	2.88	0.00

*Type: LFW = low frequency words, PW = pseudowords. Cue: V = valid, I = invalid. Visual Field (VF): L = left.*

In order to assess the significance of the main effects and interactions, we performed Type III test (which is based on control sum coding rather than dummy coding), comparing a model in which only the corresponding effect is missing with the model containing the effect. The *p*-values were calculated via the likelihood ratio tests. The type of string main effect was significant χ^2^(2) = 80.42, *p* < 0.0001, indicating that the accuracy was different for the three types of string. The spatial cue main effect was significant, χ^2^(2) = 6.83, *p* < 0.05, indicating that accuracy was modulated by the spatial cue. The visual field main effect was significant, χ^2^(1) = 353.86, *p* < 0.0001, indicating that that accuracy was better in the right visual field than in the left visual field. The interaction type of string by spatial cue was significant, χ^2^(4) = 16.51, *p* < 0.01, indicating that the effect of the spatial cue was different for the three types of string. The interaction visual field by spatial cue was not significant, χ^2^(2) = 3.30, *p* = 0.19, indicating that the effect of the spatial cue was similar in the two hemifields. The interaction type of string by visual field was not significant, χ^2^(2) = 4.61, *p* = 0.09, indicating that the effect of the type of string was similar in the two hemifields. However, the three-way interaction just missed significance, χ^2^(4) = 9.12, *p* = 0.05, suggesting that the effect of the spatial cue on the types of string was different in the two hemifields for at least one of the three types.

**FIGURE 2:**
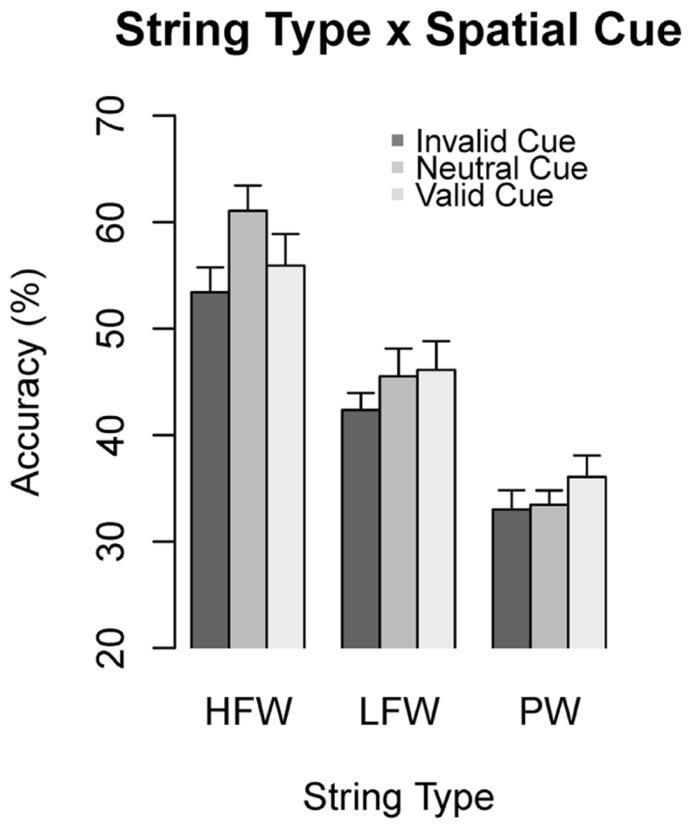
**Accuracy in string identification (percentage of correctly reported letters) as a function of type of string and validity of the spatial cue.** Error bars represent standard error of the means (SEMs).

**FIGURE 3:**
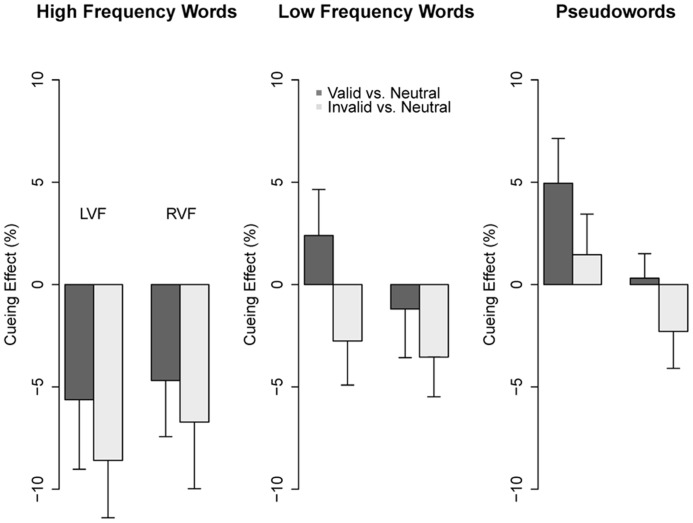
**Cueing effect (in percentage) for the three types of string, using neutral condition as baseline.** This was computed as difference between valid and neutral condition (dark gray), and difference between invalid and neutral condition (light gray). LVF, left visual field; RVF, right visual field. Error bars represent SEMs.

The interaction between type of string and spatial cue, which is crucial for the purpose of the present study, is shown in **Figure 2**. The nature of this interaction was inspected conducting separate multilevel models on each level of the *type of string* factor. Hence, for this analysis the main effect and the interaction term of the type of string were excluded. In addition, since in the full model the interaction type of string × spatial cue × visual field just missed significance, we first assessed for each model (i.e., type of string) whether inclusion of the *visual field* by *cue* interaction would improve the model fit according to the likelihood ratio tests. This was the case only for PWs (HFW: χ^2^(2) = 2.46, *p* = 0.29; LFW: χ^2^(2) = 0.39, *p* = 0.82; PW: χ^2^(2) = 7.20, *p* < 0.05). Therefore, for HFWs and LFWs the visual field factor was excluded. Factors were dummy coded with valid or neutral cue as reference levels. We report regression coefficients (*b*), *z* and *p* values. **Figure 3** shows how accuracy for each type of string changed as a function of cue condition and hemifield, using the neutral cue as baseline.

For PWs in the right visual field, accuracy did not significantly differ across cue conditions (valid vs. invalid: *b* = -0.05, *z* = -1.04, *p* = 0.30; valid vs. neutral: *b* = -0.01, *z* = -0.16, *p* = 0.87; invalid vs. neutral: *b* = -0.06, *z* = -1.16, *p* = 0.25). For PWs in the left visual field, accuracy was significantly higher in the valid condition in comparison to both the invalid and the neutral condition (respectively *b* = -0.16, *z* = -2.30, *p* < 0.05 and *b* = -0.23, *z* = -3.15, *p* < 0.01). The difference between the neutral and the invalid conditions was not significant (*b* = 0.07, *z* = 0.91, *p* = 0.36). For LFWs, none of the effects reached significance (valid vs. invalid: *b* = -0.07, *z* = -1.64, *p* = 0.10; valid vs. neutral: *b* = -0.02, *z* = -0.43, *p* = 0.66; neutral vs. invalid: *b* = -0.05, *z* = -1.35, *p* = 0.18). Finally, for HFWs, there was no difference between valid and invalid conditions (*b* = -0.04, *z* = -1.14, *p* = 0.25). However, the neutral condition showed higher accuracy than both the valid condition (*b* = -0.09, *z* = -2.62, *p* < 0.01) and the invalid condition (*b* = -0.13, *z* = -4.20, *p* < 0.001).

## DISCUSSION

The central question addressed in the present study is how spatial attention affects the processing of visual words. To this end, in the context of a perceptual identification paradigm, we manipulated the focus of attention concurrently with the type of string. HFWs, LFWs, and PWs were presented in parafoveal view, either in the left or in the right visual field. Target strings were preceded by a spatial cue that oriented attention to the target location (valid condition) or away from it (invalid condition). In the neutral condition, the cue broadened the focus of attention by directing it on both possible locations. The results of previous studies using various variants of the cueing paradigm do not offer a clear and uncontroversial picture. A novel aspect of our study was the control of random variability both at the subject and items level by exploiting mixed-effects models (Baayen et al., 2008), thereby increasing the sensitivity of the analyses and eliminating confounding factors that might affect the results.

Performance was markedly superior in the right visual field than in the left visual field, in agreement with previous studies that found a right visual field advantage for briefly presented parafoveal words (e.g., Mishkin and Gorgays, 1952; Ducrot and Grainger, 2007; Siéroff and Riva, 2011). The direct access to the left hemisphere for right presented word, scanning reading habits and attentional effects are the different factors most likely involved in the emergence of a right visual field superiority effect (see Siéroff et al., 2012, for further discussion).

Performance was also significantly affected by the spatial cue, but crucially it varied with the type of string (see **Figure 2**). In addition, but for PWs only, the cueing effect was modulated by the visual field (see **Figure 3**). In particular, PW identification was affected by the spatial cue when the string was presented in the left visual field, in agreement with previous studies that found a larger cueing effect in the left visual field (Nicholls and Wood, 1998; Gatheron and Siéroff, 1999). PWs were better identified in the valid condition, that is when attention was focused on the target location. For LFWs, the spatial cue effect was not significant but the mean accuracies showed a similar trend. These results are consistent with those of Sieroff and Posner (1988), Auclair and Siéroff (2002), as well as with the assumption of the CDP+ model (Perry et al., 2007) that the phonological route implies parsing of the string into sub-lexical units by sweeping the attentional focus from left to right across letters. Therefore, the pre-allocation of spatial attention to the target position following a valid cue meets the processing demands of phonological decoding and PW processing in particular, in line with previous studies that have linked spatial attention to phonological decoding (e.g., Facoetti et al., 2006, 2010; Ruffino et al., 2010). This explanation is also supported by the significant interaction between spatial cue and visual field for PWs. The attentional bias theory (Kinsbourne, 1970) assumes that more attentional resources are allocated to the right visual field. Accordingly, a valid cue will be more effective for the location where the least amount of attention is already allocated (Siéroff et al., 2012). This implies that the processing of stimuli that require more attention will exhibit a greater advantage.

A completely different pattern emerged for HFWs. Strikingly, word identification was best in the neutral cue condition that is when attention was directed to both the possible locations. The neutral condition showed an advantage with respect to both the valid and the invalid conditions. Given that the lateralized cues were uninformative of target location, it could be argued that the unexpected advantage of neutral trials might reflect a form of inhibition of return (Klein, 2000) that follows the exogenous shift to the lateral locations. However, this interpretation falls short in explaining why the advantage of neutral trials would be limited to the HFWs. Indeed, the classic time course of inhibition of return leads to the prediction that the effect would be maximal for the more difficult stimuli, that is the PWs. A more plausible interpretation of this finding can be found by carefully examining the nature of the neutral cue. Indeed, the neutral cue consisted in a string of hash marks that had double length with respect to the target because it was designed to cover both the possible target locations. This implies that the cue modulated also the size of the focus of attention, as suggested by studies showing that the size of the attentional focus is automatically adjusted to the size of the cue (e.g., Eriksen and St. James, 1986; Turatto et al., 2000; Ronconi et al., 2014). Thus, in the neutral condition, attention was spread out over a portion of the visual field that was approximately twice the target string length. What is the consequence of this broader focus for the processing of visual words? *Processing gradient* models of eye movements, such as SWIFT (Engbert et al., 2002, 2005; Schad and Engbert, 2012) assume that allocation of attention can extend over fixated word to support parallel processing of several words at a time. When the orthographic stimulus is not familiar, as in LFW processing or PW decoding, the foveal load is high and the perceptual span (i.e., the visual region of effectively processed information) includes only the fixated word. In contrast, processing familiar stimuli like HFWs implies low foveal load and therefore a wider perceptual span that extends over several neighboring words (e.g., LaBerge and Brown, 1989; Henderson and Ferreira, 1990). The notion that the size of the attentional window during visual word processing might be broader than the length of target words is also supported by the eye movements literature (e.g., Kennedy and Pynte, 2005; Kliegl et al., 2006, 2007; Wang et al., 2009; Dare and Shillcock, 2013; Kennedy et al., 2013) and by the finding that lateral information can affect the processing of a centrally presented target as a function of its familiarity (e.g., Lee and Kim, 2009; Waechter et al., 2011; Khelifi et al., 2012).

Therefore, HFWs, due to their overlearned representation, can provide a strong feedback signal toward lower areas of the visual system allowing fast identification of the string. The low perceptual load, due to this stronger top-down support, allows the distribution of attentional resources on a broader region of space (e.g., Brand-D’Abrescia and Lavie, 2007; see for a review Lavie, 2005). Notably, this top-down support might also compensate for the slower bottom-up processing implied by a broader focus of attention (as assumed in the zoom-lens model).

Given that a broad distribution of attention appears to be the default mode during processing of HFWs (e.g., Kliegl et al., 2006; Brand-D’Abrescia and Lavie, 2007; Schad and Engbert, 2012; Ghahghaei et al., 2013), it is conceivable that the identification of HFWs in the present study was better in the neutral condition because the cue triggered a broader attentional focus. Indeed, the attention literature shows that optimal performance in perceptual identification is obtained with an adequate allocation of attentional resources and that too much focused attention may be not beneficial (Yeshurun and Carrasco, 1998). Focused spatial attention is necessary to obtain spatial detail (e.g., Yeshurun and Carrasco, 1999; Ho et al., 2002; Hochstein and Ahissar, 2002; see Anton-Erxleben and Carrasco, 2013 for a review), whereas recognition of HFWs might be facilitated by a more global processing. Dehaene et al. (2005) suggested a neuronal model of word recognition that, in order to solve the problem of location and size invariance, postulates increasingly broader and more abstract local combination detectors (LCD model). Written words are encoded by a hierarchy of neurons with increasingly larger receptive fields, successively tuned to increasingly complex word fragments (McCandliss et al., 2003; Dehaene et al., 2005; Dehaene and Cohen, 2011). At the highest levels of this hierarchy, detectors presumably are responsive to whole words and their broad receptive field allow to respond with spatial invariance across a large part of the visual field (also see Di Bono and Zorzi, 2013). HFWs have an overlearned orthographic representation, probably located in the left ventral occipito-temporal cortex, the “visual word form area” (e.g., Glezer et al., 2009 see Dehaene and Cohen, 2011).

Although the previous data of Ducrot and Grainger (2007) brought to different conclusions, three main differences between their study and ours might explain the discordant findings. First, target duration in their study was 30 ms shorter (i.e., 50 vs. 80 ms). The deployment of attention along the whole letter string is a process that takes time (Ghahghaei et al., 2013). Therefore, it is possible that 50 ms of target duration are not enough to detect fine modulations of the attentional focus. Benso et al. (1998) studied the time course of attentional focusing with a standard spatial cue-size paradigm. While they showed that the focus of attention requires 33–66 ms to adjust to object size in the fovea, they found that the control of the attentional focus in the periphery took place only when the interval between the cue and the stimulus was between 300 and 400 msec. Summing together cue duration, delay time and target duration in our paradigm results in an overall time of 160 ms during which the size of the focus might be modulated, an intermediate value that seems suitable for our parafoveal stimuli. A second difference is that the stimuli of Ducrot and Grainger (2007) did not include PWs. There is growing evidence that reading is context dependent even at the single word level (e.g., Reynolds and Besner, 2005; O’Malley and Besner, 2008; Reynolds et al., 2010). For example, O’Malley and Besner (2008) showed that the presence of PWs in the list composition changed the effect of stimulus degradation on the modulation of the frequency effect. In the same vein, it seems likely that the presence of PWs in our study promoted a more flexible shaping of the attentional focus. Finally, the cuing paradigm of Ducrot and Grainger (2007) did not include the invalid condition. The presence of an invalid condition in our study is likely to have induced a stronger cueing effects and in turn a more effective modulation of the deployment of spatial attention. It could be argued that the lateralized spatial cues in Ducrot and Grainger’s (2007) study were highly informative because they perfectly predicted the location of the letter string (unlike our study, in which they were uninformative). However, it is unlikely that this discrepancy implies a different type of attentional orienting, because cue-target stimulus onset asynchrony (SOA) in their study was too short (i.e., 80 ms) to allow voluntary deployment. That is, attention orienting was stimulus-driven both in their study and in ours.

In conclusion, we found that the manipulation of spatial attention affects string processing and this influence was modulated by the type of string, as predicted by the CDP+ model of reading (Perry et al., 2007) as well as by processing gradient models (e.g., LaBerge and Brown, 1989; Henderson and Ferreira, 1990; Schad and Engbert, 2012). Processing of unfamiliar strings, such as LFW and PW, is affected by directing attention to a different location and it is facilitated by attentional focusing. Conversely, identification of HFWs was enhanced in a condition promoting distributed attention, an attentional set that appears to be the default mode during reading of familiar words and is likely to optimally engage the broad receptive fields of the highest detectors in the hierarchical system for visual word recognition. However, the explanation of this novel finding is speculative and it therefore warrants further investigation.

## Conflict of Interest Statement

The authors declare that the research was conducted in the absence of any commercial or financial relationships that could be construed as a potential conflict of interest.
